# Polycystic Ovary Syndrome: Novel and Hub lncRNAs in the Insulin Resistance-Associated lncRNA–mRNA Network

**DOI:** 10.3389/fgene.2019.00772

**Published:** 2019-08-22

**Authors:** Jun Zhao, Jiayu Huang, Xueying Geng, Weiwei Chu, Shang Li, Zi-Jiang Chen, Yanzhi Du

**Affiliations:** ^1^Center for Reproductive Medicine, Ren Ji Hospital, School of Medicine, Shanghai Jiao Tong University, Shanghai, China; ^2^Shanghai Key Laboratory for Assisted Reproduction and Reproductive Genetics, Shanghai, China; ^3^Center for Reproductive Medicine, Shandong Provincial Hospital, Shandong University, National Research Center for Assisted Reproductive Technology and Reproductive Genetics, The Key Laboratory for Reproductive Endocrinology, Ministry of Education, Shandong Provincial Clinical Medicine Research Center for Reproductive Health, Shandong Provincial Key Laboratory of Reproductive Medicine, China

**Keywords:** insulin resistance, polycystic ovary syndrome, type 2 diabetes, metabolic and reproductive disorder, long non-coding RNAs, lncRNA protein interactions, insulin resistance-associated lncRNA–mRNA network

## Abstract

Polycystic ovary syndrome (PCOS) is a common metabolic and reproductive disorder with an increasing risk for type 2 diabetes. Insulin resistance is a common feature of women with PCOS, but the underlying molecular mechanism remains unclear. This study aimed to screen critical long non-coding RNAs (lncRNAs) that might play pivotal roles in insulin resistance, which could provide candidate biomarkers and potential therapeutic targets for PCOS. Gene expression profiles of the skeletal muscle in patients with PCOS accompanied by insulin resistance and healthy patients were obtained from the publicly available Gene Expression Omnibus (GEO) database. A global triple network including RNA-binding protein, mRNA, and lncRNAs was constructed based on the data from starBase. Then, we extracted an insulin resistance-associated lncRNA–mRNA network (IRLMN) by integrating the data from starBase and GEO. We also performed a weighted gene co-expression network analysis (WGCNA) on the differentially expressed genes between the women with and without PCOS, to identify hub lncRNAs. Additionally, the findings of key lncRNAs were examined in an independent GEO dataset. The expression level of *lncRNA RP11-151A6.4* in ovarian granulosa cells was increased in patients with PCOS compared with that in control women. Levels were also increased in PCOS patients with higher BMI, hyperinsulinemia, and higher HOMA-IR values. As a result, *RP11-151A6.4* was identified as a hub lncRNA based on IRLMN and WGCNA and was highly expressed in ovarian granulosa cells, skeletal muscle, and subcutaneous and omental adipose tissues of patients with insulin resistance. This study showed the differences between lncRNA and mRNA profiles from healthy women and women with PCOS and insulin resistance. Here, we demonstrated that RP11-151A6.4 might play a vital role in insulin resistance, androgen excess, and adipose dysfunction in patients with PCOS. Further study concerning RP11-151A6.4 could elucidate the underlying mechanisms of insulin resistance.

## Introduction

Polycystic ovary syndrome (PCOS) is a common endocrine and metabolic disorder, defined by a combination of symptoms related to hyperandrogenism and ovarian dysfunction, upon exclusion of other diagnoses. The prevalence of PCOS ranges from 6% to 20% in women of reproductive age (Ricardo Azziz et al., 2016; Escobar-Morreale, 2018). PCOS is associated with reproductive co-morbidities (menstrual irregularity, ovulatory dysfunction, infertility, and pregnancy complications), metabolic disorders (metabolic syndrome, type 2 diabetes, and cardiovascular disease), and psychological risk factors (anxiety and depression).

Insulin resistance, a clinical feature of PCOS, is a decreased ability of insulin to mediate metabolic actions on glucose uptake, production, and/or lipolysis. It results in a requirement for increased amounts of insulin to achieve a given metabolic action (Miller et al., 2017). Insulin resistance and compensatory hyperinsulinism contribute to hyperandrogenemia and ovulatory disturbances of PCOS (Diamanti-Kandarakis and Dunaif, 2012), mainly because insulin facilitates androgen secretion from the pituitary, ovaries, adrenal glands, and liver (Barbieri et al., 1986; Tosi et al., 2011); acts as a co-gonadotropin to modulate ovarian steroidogenesis (Nestler et al., 1998; Diamanti-Kandarakis and Dunaif, 2012); arrests ovarian follicular development (Dumesic and Abbott, 2008); modulates luteinizing hormone pulsatility (Adashi et al., 1981); and negatively regulates sex hormone binding globulin production (Nestler et al., 1991). Decreasing insulin resistance through insulin-sensitizing drugs (e.g., metformin, rosiglitazone, pioglitazone, and d-chiro-inositol) and/or lifestyle treatments could improve the reproductive and metabolic outcomes for patients with PCOS (Morley et al., 2017; Anithasri et al., 2019). Although insulin resistance has been well-recognized in PCOS, some observations suggest that lean patients with PCOS may have normal insulin sensitivity (Lim et al., 2019). Moreover, the molecular mechanism of insulin resistance in PCOS remains unknown.

Long non-coding RNAs (lncRNAs) are non-coding RNAs with transcripts greater than 200 nucleotides in length. These lncRNAs are involved in many biological processes: transcriptional and post-transcriptional regulation; recruitment of epigenetic modifiers; control of mRNA decay; and sequestration of transcription factors (Quinn and Chang, 2016; Goyal et al., 2018). The overexpression, deficiency, or mutation of lncRNA genes has been implicated in numerous human diseases including PCOS. For instance, results from RNA sequencing data analysis showed that lncRNAs were differentially expressed in the human follicular fluid of woman with and without PCOS. lncRNAs associated with the metabolic process were highly enriched in the follicular fluid of mature follicles from the PCOS group compared with the healthy group (Jiao et al., 2018). LINC-01572:28 (Jun et al., 2018) and lncRNA HCG26 (Wang et al., 2017) were reported as regulators of cell growth and steroidogenesis in the granulosa cells of PCOS. However, few studies have identified the relationship between lncRNAs and PCOS with insulin resistance by the high throughput methods, including microarray and RNA-seq.

Therefore, to explore the potential pathogenetic role of lncRNAs in patients with PCOS and insulin resistance, we constructed a global triple network including RNA binding protein, mRNA, and lncRNAs. We extracted an insulin resistance-associated lncRNA–mRNA network (IRLMN) by integrating data from starBase and Gene Expression Omnibus (GEO). Then, we mapped the differentially expressed lncRNAs and mRNAs into the global triple network. To confirm our results, we identified hub lncRNAs using weighted gene co-expression network analysis (WGCNA) on the differentially expressed genes (DEGs) between the PCOS and healthy groups.

Our study reliably provided bunches of lncRNAs related to PCOS with insulin resistance and indicated that lncRNA RP11-151A6.4 might play an important role in the insulin resistance. Thus, we provide a novel perspective to elucidate the underlying mechanisms of insulin resistance in patients with PCOS.

## Materials and Methods

### lncRNA–RBP and RBP–mRNA Interactions

We obtained interaction data from starBase v3.0 (http://starbase.sysu.edu.cn/). starBase is an open-source platform for exploring the RNA-binding protein (RBP)–lncRNA and RBP–mRNA interactions from multidimensional sequencing data. We downloaded 17,604 lncRNA–RBP interaction pairs and 207,443 mRNA–RBP interactions, including 6238 lncRNAs, 33 RBPs, and 17,855 mRNAs.

### Gene Expression Profile

We searched the associated gene expression profiles of PCOS with insulin resistance in GEO (https://www.ncbi.nlm.nih.gov/geo/), using the keywords “PCOS,” “insulin resistance,” and “Homo sapiens.” We retained gene expression datasets from the GeneChip™ Human Genome U133 Plus 2.0 Array (HG-U133) (Thermo Fisher Scientific, Waltham, MA, USA). Considering the quality of data and sample size, we chose GSE8157 (Skov et al., 2008) for further analysis. We detected gene expression changes in the skeletal muscles of 10 patients with PCOS and 13 healthy women (control). We also explored the effect of pioglitazone (30 mg/day for 16 weeks) on gene expression in the skeletal muscle of 10 obese women with PCOS who were metabolically characterized by a euglycemic-hyperinsulinemic clamp.

### Probe Re-annotation

The HG-U133 probe sequences were aligned to the human long non-coding transcript sequences and protein-coding transcript sequences from the GENCODE database (https://www.gencodegenes.org), using the Basic Local Alignment Search Tool (BLAST^®^) nucleotide–nucleotide (BLASTn) program. The results from the sequence alignment were filtered:

1. each transcript should be perfectly matched to more than four probes;
2. a set of probes can only be matched to one transcript; and
3. if the probe can be matched to more than one transcript, then it will be deleted.

### Identifying DEGs

After the GSE8157 data from GEO were downloaded, it was normalized *via* “affy” package in R software. Then, significance analysis of microarrays (SAM) was applied to analyze the two phenotypes in each cohort (PCOS and control, and PCOS before and after pioglitazone treatment) to discover DEGs. This process was accomplished *via* “siggenes” package in R. A *p*-value < 0.01 was considered statistically significant. Then, we used the R package “VennDiagram” to describe the relationship between these two sets of DEGs.

### Hyper-Geometric Test

We extracted the IRLMN from the global triple network by performing a hypergeometric test. A *p*-value < 0.01 was considered statistically significant:

$$P=1-\sum_{t=0}^{r-1}\frac{\left(\frac{t}{i}\right)\left(\frac{m-t}{n-i}\right)}{\left(\frac{m}{n}\right)}$$

where *m* represents the total RBPs; *t* represents the number of RBPs interacting with the mRNA; *n* represents the number of RBPs interacting with the lncRNA; and *r* represents the number of RBPs shared between mRNA and lncRNA.

### Module Analysis lncRNA and mRNA Interactions

To investigate the cross-talks between mRNAs and lncRNAs, we performed bidirectional hierarchical clustering on the IRLMN using R package “pheatmap.”

### WGCNA

To verify the potential functional lncRNAs that we found above, we used another method to analyze the DEGs in the muscle of patients with PCOS and insulin resistance. WGCNA, an R package for weighted correlation network analysis (Langfelder and Horvath, 2008), can be used for finding clusters (modules) of highly correlated genes and identifying candidate biomarkers or therapeutic targets. It assigns a connection weight to each gene pair in the network and uses a soft threshold, which is biologically more meaningful than are traditional methods that use binary information (0 = unconnected, 1 = connected) (Zhang and Horvath, 2005). We used the step-by-step network construction with a soft threshold of β = 15 (*R* ^2^ = 0.75) and a minimum module size of 30. The topological overlap distance, calculated from the adjacency matrix, was clustered with the average linkage hierarchical clustering. The default minimum cluster merge height of 0.10 was retained.

### Enrichment Analysis

Gene ontology (GO) analysis was used to identify the possible molecular function and visualize the potential biological meaning behind large list genes. DAVID 6.7 provided a comprehensive set of tools for this (https://david.ncifcrf.gov/). The biology process terms with a *p*-value < 0.05 were considered statistically significant.

We used the Kyoto Encyclopedia of Genes and Genomes (KEGG) database (http://www.genome.ad.jp/kegg/) to analyze the potential functions of these genes. Genes were enriched in the KEGG pathways by applying the “SubpathwayMiner” package in R (Li et al., 2009).

### Subjects

Ovarian granulosa cell samples were collected from patients who underwent *in vitro* fertilization or intracytoplasmic sperm injection at the center for Reproductive Medicine, Ren Ji Hospital, School of Medicine, Shanghai Jiao Tong University. The study was approved by the ART Ethics Committee of Ren Ji Hospital, School of Medicine, Shanghai Jiao Tong University (number 2017041411), and informed consent was obtained from all participants. The diagnosis of PCOS was based on the revised Rotterdam diagnostic criteria for PCOS (Rotterdam, 2004). All patients in the control group had regular menstrual cycles (26 to 34 days) and normal ovarian morphology by ultrasound examination. Anthropometric variables, such as age, body mass index (BMI), and select endocrine and biochemical parameters, were recorded and are presented in **Table 1**. The WHO cutoff point for normal (BMI 18.5–23.9 kg/m^2^), overweight (BMI ≥ 24 kg/m^2^), and obesity (BMI ≥ 29 kg/m^2^) for Asian populations (WHO Expert Consultation, 2004). The homeostasis model assessment of insulin resistance (HOMA-IR) index was calculated according to the following formula (Matthews et al., 1985): fasting insulin (mIU/L) * fasting glucose (mmol/L)/22.5; and insulin resistance was defined as HOMA-IR within the higher tertile. Follicle-stimulating hormone (FSH), luteinizing hormone (LH), testosterone (T), anti-Müllerian hormone (AMH), fasting glucose, and fasting insulin levels were determined using either chemiluminescent assay (Roche Diagnostics, Indianapolis, IN, USA) or ELISA (Kangrun, Guangzhou, China) kits.

**Table 1:** Background information of patients with polycystic ovary syndrome (PCOS) and controls.

	PCOS (*n* = 52)	Control (*n* = 42)	*p* value
Age (years)	28.23 ± 3.47	28.83 ± 2.86	0.345
BMI (kg/m^2^)^a^	25.92 ± 3.58	22.19 ± 2.92	<0.0001
Basal FSH (IU/L)^b^	5.59 ± 1.00	6.30 ± 1.37	<0.01
Basal LH (IU/L)^a^	9.64 ± 4.57	5.12 ± 1.92	<0.0001
Basal T (ng/dL)^a^	42.28 ± 21.50	20.06 ± 6.95	<0.0001
AMH (ng/ml)^a^	9.74 ± 4.15	5.09 ± 3.13	<0.0001
Fasting glucose (mmol/L)^c^	5.59 ± 0.52	5.35 ± 0.35	0.022
Fasting insulin (mIU/L)^b^	20.57 ± 16.71	9.31 ± 3.53	<0.01
HOMA-IR^a^	4.92 ± 4.27	1.70 ± 1.23	<0.0001

All data are means ± SD values. ^a^p < 0.001; ^b^p < 0.01; ^c^p < 0.05. FSH, follicle-stimulating hormone; LH, luteinizing hormone; T, testosterone; AMH, anti-Müllerian hormone.

### RNA Extraction and Quantitative Real-Time Polymerase Chain Reaction

Total RNA from patients’ ovarian granulosa cells was extracted by TRIzol (Invitrogen) and reverse transcribed into cDNA (PrimeScript™ RT reagent Kit, Takara, Dalian, China). Target gene expression was detected with the use of quantitative real-time polymerase chain reaction (qRT-PCR). The relative expression of RNA was calculated using the formula 2^−ΔΔCt^. β-Actin was employed as an internal control for the quantification of target genes. The ΔCt normalization was to β-actin. The primer sequences of the tested genes (RP11-151A6.4) are shown in **Supplemental Table S1**.

### Statistical Analysis

All calculations were performed using the SPSS statistical software package (version 20.0) (IBM Corp, Armonk, NY, USA). Results were expressed as the mean ± standard deviation (SD). The Kolmogorov–Smirnov test was used to assess whether the data were of normal distribution. Statistical comparisons between two variables (the PCOS group and the control group) were performed using the Student *t*-test for normal distribution, and unpaired *t*-test and the Mann–Whitney *U* test were used for continuous variables. Data were considered statistically significant for *p* < 0.05. Statistical significance was evaluated using data from at least three independent experiments.

## Results

### Differentially Expressed lncRNAs and mRNAs Associated With Insulin Resistance *via* Probe Re-annotation

**Figure 1:**
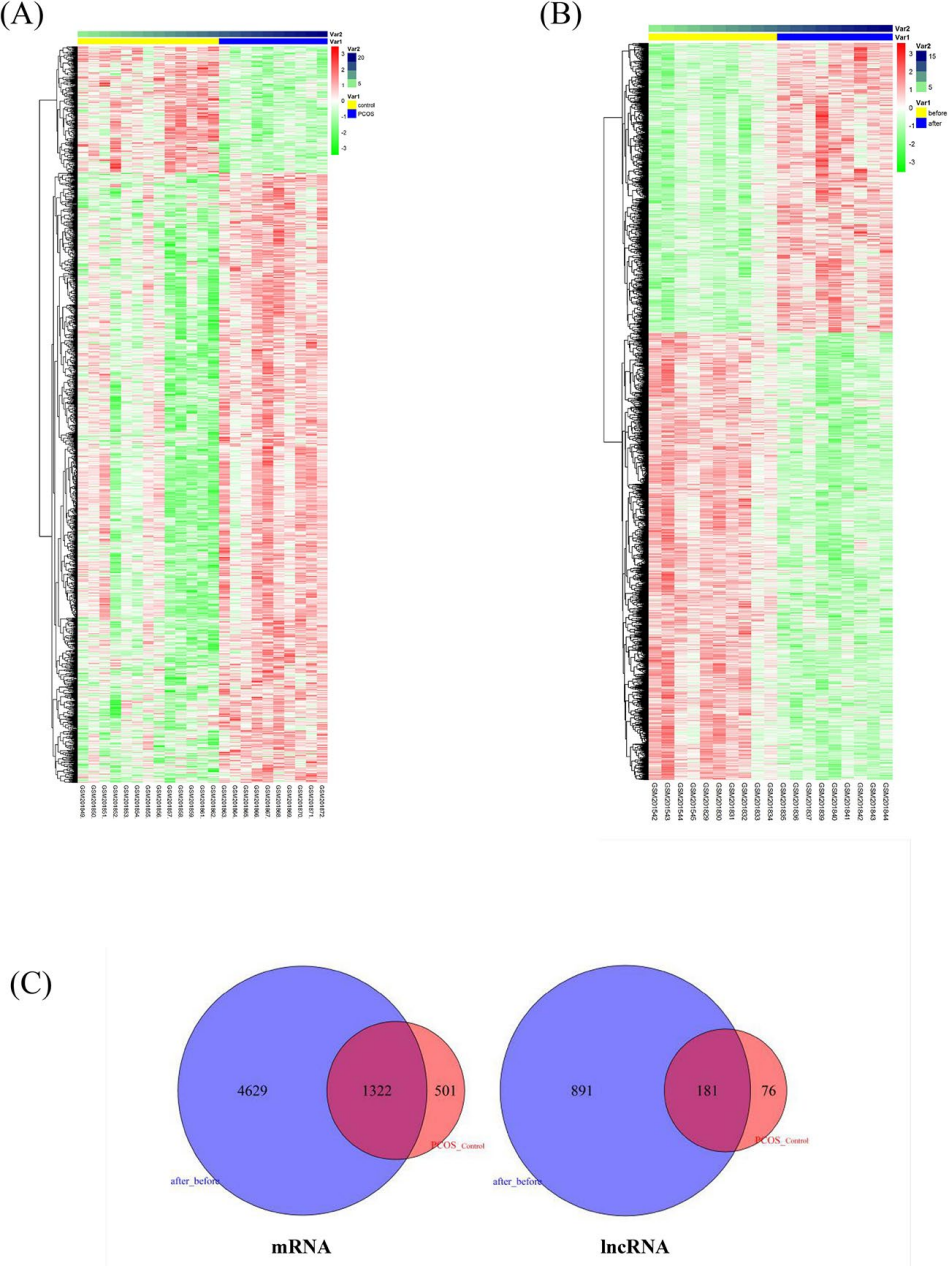
Differentially expressed lncRNAs and mRNAs associated with insulin resistance in patients with polycystic ovary syndrome (PCOS). **(A)** Heatmap of differentially expressed transcripts in the muscle of patients with PCOS and insulin resistance, compared with levels in controls. The columns represent 10 patients with PCOS (Var1, blue columns) and 13 healthy women (Var1 yellow columns). Var2 represents the number of samples. The rows represent lncRNAs and their mRNA neighbors. The red and green rows are based on expression in different samples. Red rows indicate upregulation, whereas green rows indicate downregulation. The darker the color, the greater the difference. **(B)** Heatmap of significantly changed transcripts after pioglitazone treatment. The columns represent 10 obese women with PCOS before (Var1 yellow columns) and after pioglitazone treatment (Var1 blue columns). Var2 represents the number of samples. **(C)** Venn diagram of commonly identified lncRNAs and mRNAs in both dataset 1 (PCOS and control) and dataset 2 (before and after treatment); 181 and 1,322 similar lncRNAs and mRNAs were identified respectively and then mapped into the global triple network.

We applied a sequence alignment algorithm between the probe sequences and human long non-coding transcript sequences and protein-coding transcript sequences *via* BLAST^®^. We re-annotated HG-U133 probes. From the two annotation sets, we identified 4,837 lncRNA–probe pairs and 33,846 mRNA–probe pairs. We calculated the two GSE8157 datasets using SAM. A total of 2,290 transcripts were differentially expressed in the muscle of patients with PCOS and insulin resistance, compared with the control (**Figure 1A**); and 8,596 transcripts changed after pioglitazone treatment in patients with PCOS (**Figure 1B**). A total of 181 and 1,322 similar lncRNAs and mRNAs, respectively, were identified in both datasets 1 (PCOS and control) and 2 (before and after treatment) (**Figure 1C**). For the further explanation, we mapped the 181 lncRNAs and 1,322 mRNAs in **Figure 1C** into the global triple network.

### Construction of the IRLMN From The Global Triple Network

**Figure 2:**
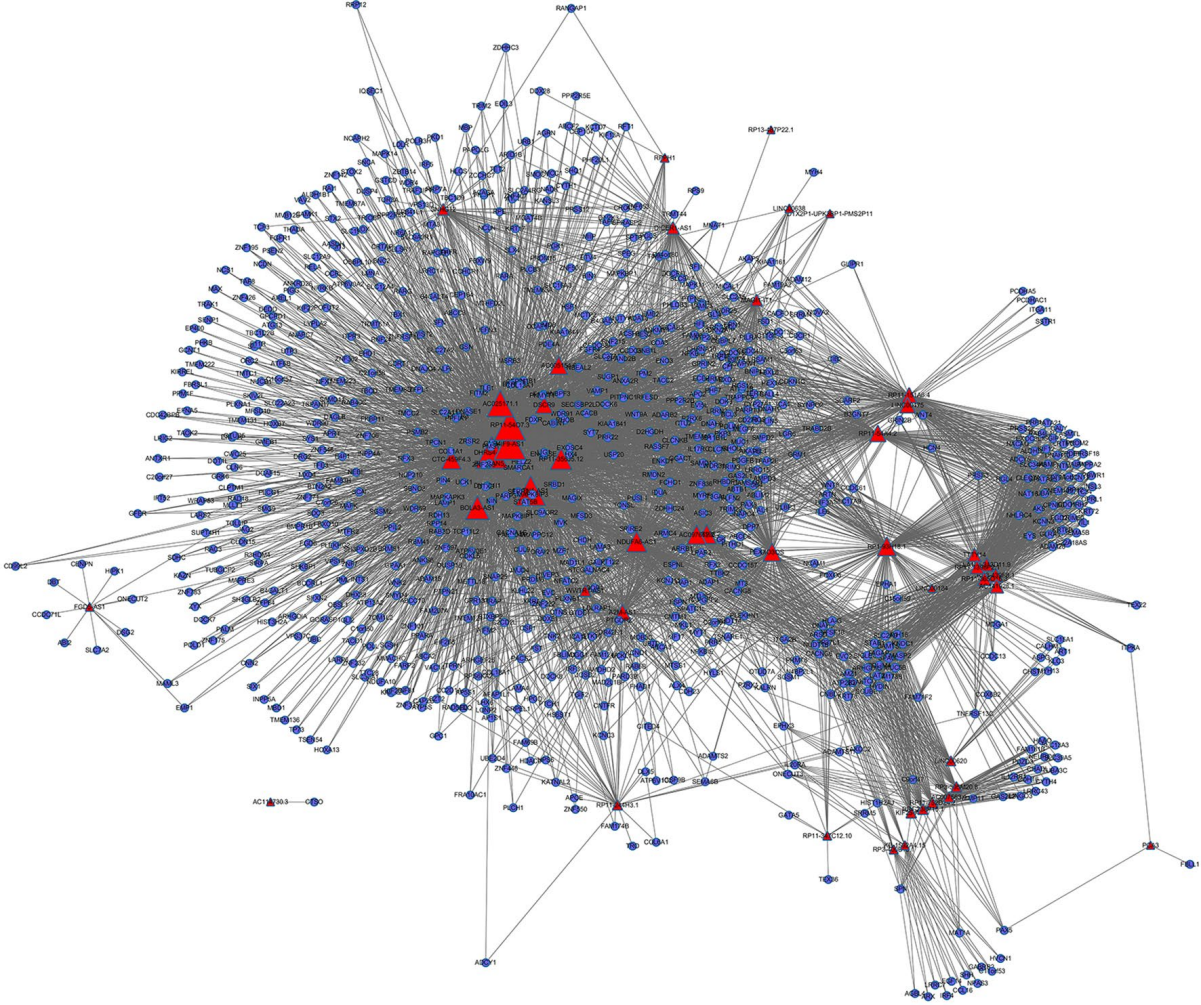
Insulin-resistance lncRNA–mRNA network (IRLMN). The blue nodes represent mRNA, and the red nodes represent lncRNAs. There were 47 lncRNA nodes, 852 mRNA nodes, and 5,705 edges in the network. The nodes with colorful labels are lncRNAs that are highly related to insulin resistance in our study. The size of the nodes represents the degrees of nodes in the network.

We identified 225,047 interaction pairs and constructed a global triple network including lncRNAs, mRNAs, and RBPs, based starBase v3.0. Then, we defined the lncRNAs, mRNAs, and RBPs as nodes in the network. The interaction between each node was evaluated by adding an edge between them. Then, we mapped the 181 lncRNAs and 1,322 mRNAs that we found above into the global triple network; and we obtained 71 mapped lncRNAs and 1,187 mapped mRNAs. We extracted these genes and their linked RBPs to new triple pairs. The significance of the number of shared RBPs between lncRNA and mRNA pairs was evaluated *via* the hypergeometric test. Finally, we constructed a novel IRLMN, containing 47 lncRNAs nodes, 852 mRNA nodes, and 5,705 edges (**Figure 2**).

### IRLMN Module Analysis

**Figure 3:**
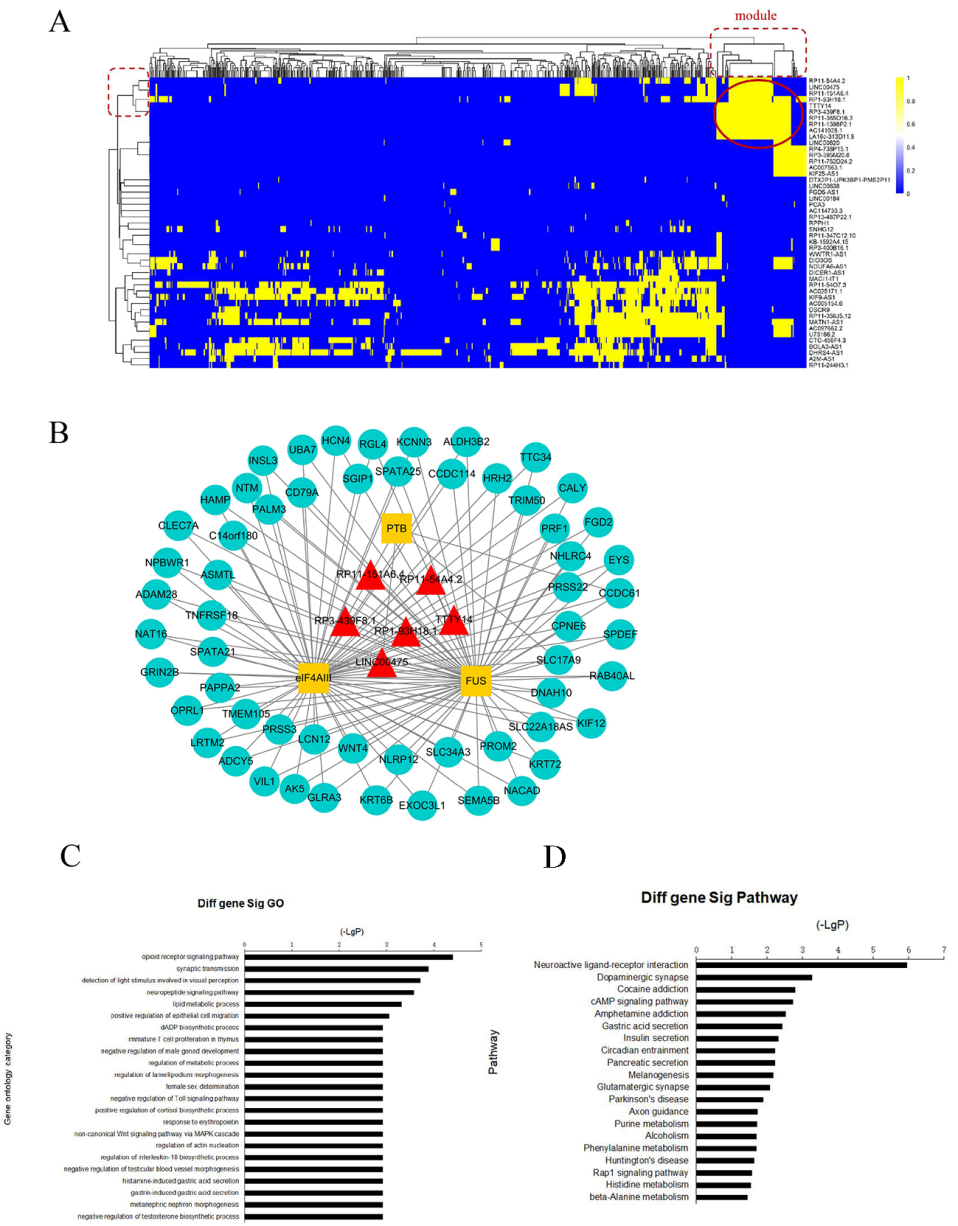
Module analysis of lncRNA and mRNA interactions. **(A)** Heatmap of insulin resistance-associated lncRNA–mRNA network (IRLMN). The bidirectional hierarchical cluster was performed with R software. The red ellipses represent the module (the same for the rectangles in the cluster tree). The color scale of blue to yellow indicates differences in correlations (from 0 to 1). The closer the color to yellow (to 1), the stronger the correlation. **(B)** Module network extracted from IRLMN, consisting of six lncRNAs (red triangles), three RNA-binding proteins (RBPs) (yellow rectangles), and 58 mRNAs (blue ellipses). **(C)** Gene ontology (GO) enrichment analysis of module; the *x*-axis is the −log10 of the *p*-value, and *p* < 0.05 was considered statistically significant. The *y*-axis shows the biological processes. **(D)** Subpathway enrichment analysis of the module; the *x*-axis is the −log10 of the *p*-value, and *p* < 0.05 was considered statistically significant. The *y*-axis shows the subpathways.

To further investigate the cross-talks between nodes, we used bidirectional hierarchical clustering on the IRLMN using the “pheatmap” package in R software (**Figure 3A**). We identified a highly related insulin resistance-associated module. It consisted of six lncRNAs (**Table 2**), three RBPs, and 58 mRNAs (**Figure 3B**). GO enrichment analysis and subpathway analysis for the genes in this module revealed that “lipid metabolic process,” “regulation of metabolic process,” and “negative regulation of Toll signaling pathway” were significant and highly related to insulin resistance (**Figure 3C**). Moreover, “cAMP signaling pathway” and “insulin secretion” were also significantly enriched in this module (**Figure 3D**).

**Table 2:** The hub lncRNAs in the module by bidirectional hierarchical clustering analysis.

Gene	Type	Binding proteins
RP11-151A6.4	lncRNA	PTB, eIF4AIII, FUS
LINC00475	lncRNA	PTB, eIF4AIII, FUS
RP11-54A4.2	lncRNA	eIF4AIII, FUS
TTTY14	lncRNA	eIF4AIII, FUS
RP1-93H18.1	lncRNA	eIF4AIII, FUS
RP3-439F8.1	lncRNA	eIF4AIII, FUS

### WGCNA Analysis of DEGs in the Muscle of Patients With and Without PCOS

**Figure 4:**
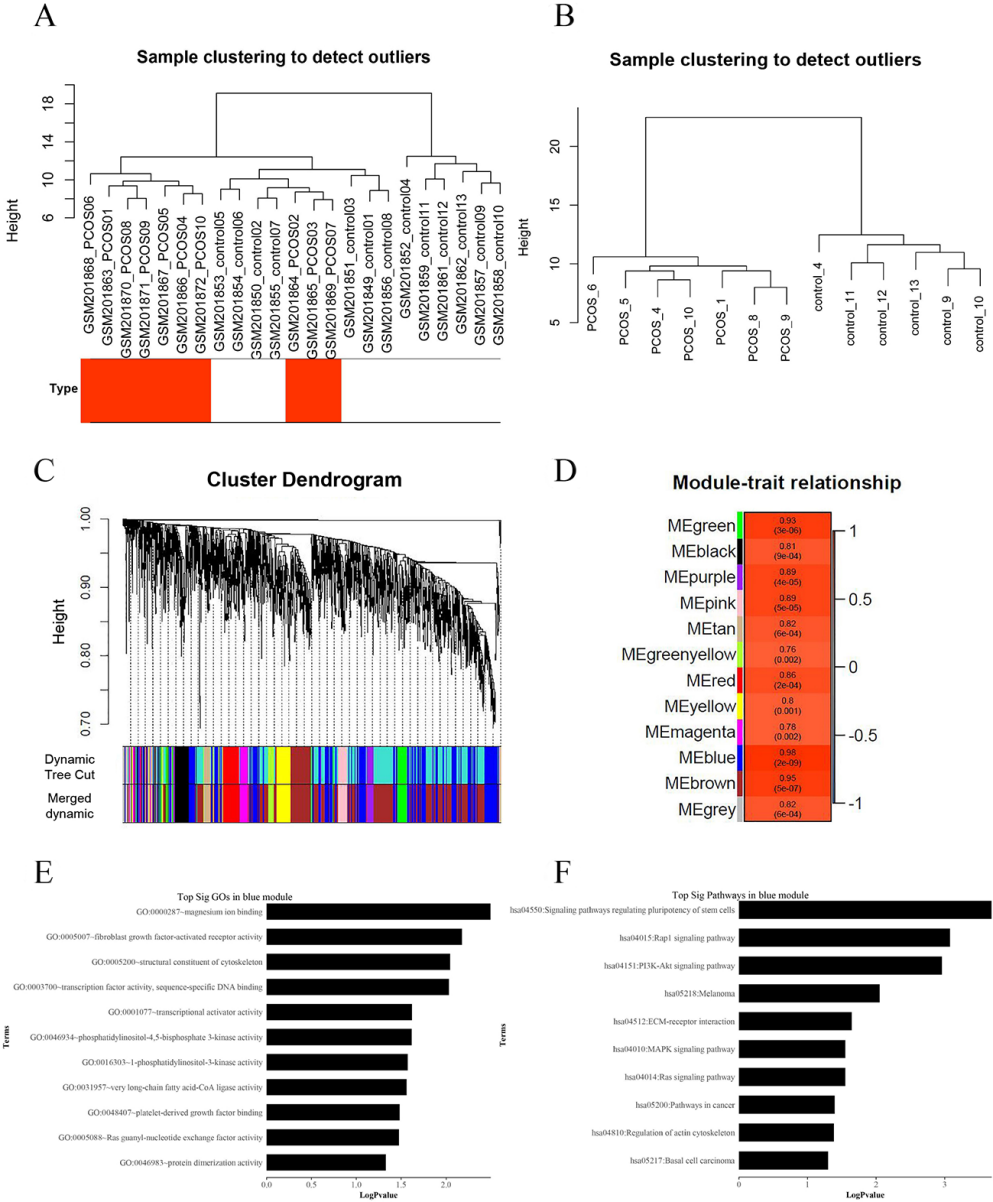
Weighted gene co-expression network analysis (WGCNA) of differentially expressed genes associated with insulin resistance in patients with polycystic ovary syndrome (PCOS). **(A)** Hierarchical clustering of 23 skeletal muscle samples (10 cases and 13 controls) from women with and without PCOS. The rows represent the types of samples. The red row represents cases, and the white row represents controls. **(B)** Hierarchical clustering of 13 samples (seven cases and six controls) after deleting the outlier samples. **(C)** Gene dendrogram obtained by average linkage hierarchical clustering. The color row underneath the dendrogram shows the module assignment determined by the Dynamic Tree Cut. **(D)** Module–PCOS relationship. The *y*-axis shows the modules. The first row of each module is the *R*^2^ value; and the closer *R*^2^ is to 1, the stronger the correlation is. The second row of each module is the *p*-value, and *p* < 0.01 was considered statistically significant. The blue module was most closely correlated with insulin resistance of PCOS (*R*^2^ = 0.98, *p*-value = 2e−09). **(E)** The gene ontology (GO) enrichment analysis of the module; the *x*-axis is −log10 of *p*-value, and *p* < 0.05 was considered statistically significant. The *y*-axis shows the biological processes. **(F)** Subpathway enrichment analysis of the module; the *x*-axis is −log10 of *p*-value, and *p* < 0.05 was considered statistically significant. The *y*-axis shows the subpathways.

We applied WGCNA to explore the potential mechanisms of insulin resistance in patients with PCOS, using DEGs in the above analysis, and to verify the possible functional lncRNAs found in IRLMN. A total of 2,290 transcripts were altered in the muscle of patients with PCOS, compared with those in the control; and these were selected for analysis. **Figure 4A** shows the cluster of 23 skeletal muscle samples from patients with and without PCOS, and 10 of them were clustered abnormally. Thus, after these abnormal samples were deleted, seven PCOS samples and six healthy control samples were used for analysis (**Figure 4B**). The blue module was most closely correlated with the insulin resistance of PCOS (*R* ^2^ = 0.98, *p* = 2e−09) (**Figures 4C, D**). The first row of each module is the *R* ^2^ value; and the closer *R* ^2^ is to 1, the stronger the correlation is. The second row of each module is the *p*-value, and *p* < 0.01 was considered statistically significant.

GO and KEGG analyses (**Figures 4E, F**) showed that the “Rap1 signaling pathway” and “PI3K-Akt signaling pathway” were highly enriched in the blue module. The DEGs in this module were mainly associated with functions like “magnesium ion binding,” “very long-chain fatty acid-CoA ligase activity,” and “phosphatidylinositol-4,5-bisphosphate 3-kinase activity.” According to the network topological index, seven hub lncRNAs (**Table 3**) were selected from the module and compared with the list of lncRNAs that we obtained in IRLMN.

**Table 3:** The top seven hub lncRNAs in blue module by WGCNA.

Name	Node	Weight	Type
LAMA5-AS1	608	243.4795	lncRNA
RP11-730N24.1	608	243.0766	lncRNA
TRPM2-AS	608	227.1972	lncRNA
LINC01770	608	214.5427	lncRNA
RP11-151A6.4	608	190.5272	lncRNA
TSPEAR-AS2	608	189.5206	lncRNA
LINC02092	608	186.322	lncRNA

### lncRNA RP11-151A6.4 May Play a Role in the Pathogenesis of Insulin Resistance in Patients With PCOS

**Figure 5:**
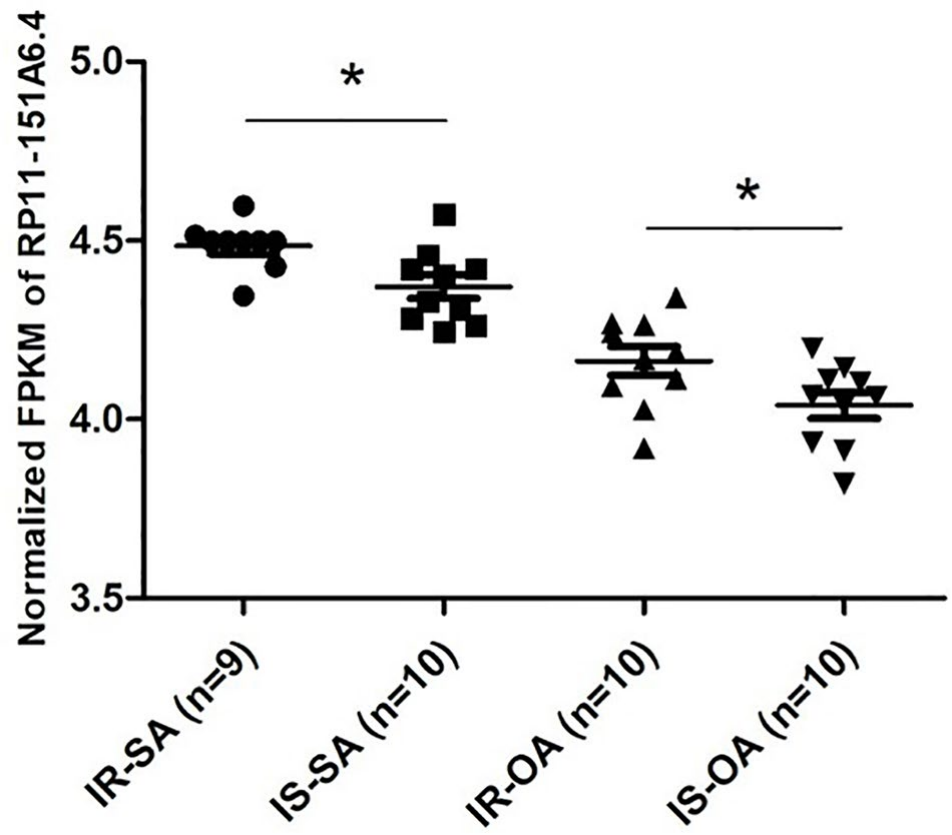
Elevated expression of lncRNA RP11-151A6.4 in the subcutaneous and omental adipose tissues of patients with insulin resistance. Expression level of RP11-151A6.4 in the subcutaneous and omental adipose tissues of people with and without insulin resistance. IR, insulin resistance; IS, insulin sensitive; SA, subcutaneous adipose tissue; OA, omental adipose tissue. *p < 0.05 and ns: not significant, *p* > 0.05, according to two-tailed Student’s *t*-test.

**Figure 6:**
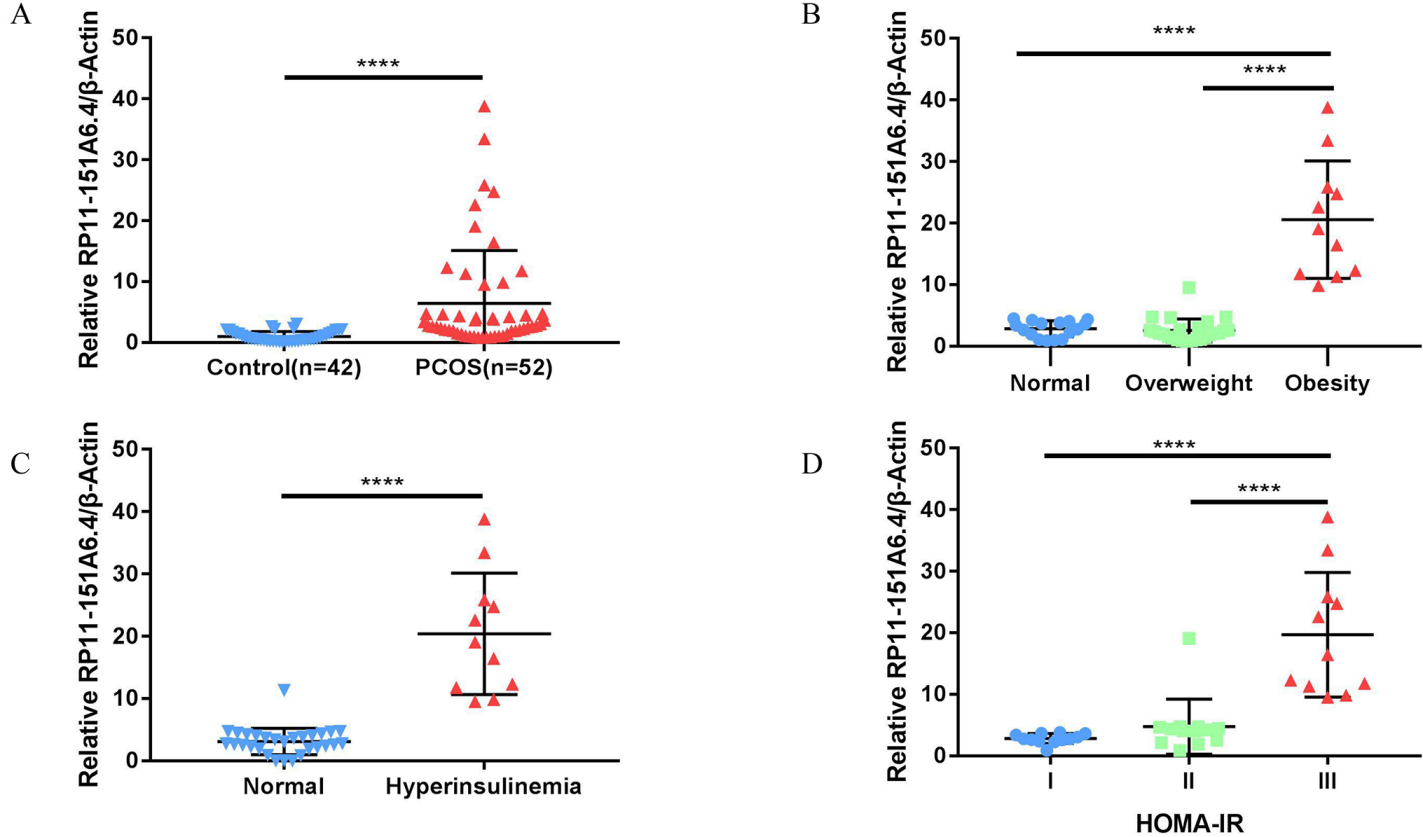
Elevated expression of lncRNA RP11-151A6.4 in the ovarian granulosa cells of patients with insulin resistance. The expression level was detected *via* qRT-PCR and normalized to β-actin levels. ****p < 0.0001, ***p < 0.001, **p < 0.01, *p < 0.05. **(A)** The expression level of RP11-151A6.4 was increased in patients with PCOS compared with control women. **(B)** The expression level of RP11-151A6.4 was correlated with body mass index (BMI) in patients with PCOS. **(C)** The expression level of RP11-151A6.4 was elevated in PCOS patients with hyperinsulinemia (fasting insulin > 23 mIU/L). **(D)** Higher HOMA-IR values were associated with higher expression levels of RP11-151A6.4. Ranges of HOMA-IR values were as follows: tertile I (0.95 to 2.79), tertile II (2.79 to 4.49), and tertile III (4.49 to 20.73).

After merging the lists of potential lncRNAs, we got through IRLMN and WGCNA, and RP11-151A6.4 was identified as a possible functional lncRNA (**Tables 2** and **3**). We examined an independent GEO dataset (GSE20950) (Hardy et al., 2011), which detected the expression data from human adipose tissues of patients with and without insulin resistance. The result showed that the expression of RP11-151A6.4 was significantly higher in both subcutaneous and omental adipose tissue of patients with insulin resistance compared with healthy patients (control) (**Figure 5**). To verify that RP11-151A6.4 is potentially involved in the etiology of PCOS with insulin resistance, we analyzed relative expression in ovarian granulosa cells in a cohort of 52 women with PCOS and 42 treatment-matched reproductively normal control women by qRT-PCR (**Figure 6**). The expression level of RP11-151A6.4 was increased in patients with PCOS compared with control women (*p* < 0.0001; **Figure 6A**). Further, levels were also increased in patients with PCOS as BMI increased (*p* < 0.001; **Figure 6B**). Moreover, levels of RP11-151A6.4 were increased in PCOS patients with hyperinsulinemia (Corkey, 2011) (fasting insulin > 23 mIU/L; *p* < 0.0001; **Figure 6C**). Moreover, ranges of HOMA-IR values in patients with PCOS were 0.95 to 2.79 for tertile I, 2.79 to 4.49 for tertile II, and 4.49 to 20.73 for tertile III (**Supplemental Table S2**). Finally, higher HOMA-IR values were associated with higher expression levels of RP11-151A6.4 (*p* < 0.0001; **Figure 6D**).

## Discussion

Here, a global triple network including lncRNAs, mRNAs, and RBPs was created using interaction data from starBase to extract the IRLMN. After bidirectional hierarchical cluster analysis, we identified an insulin resistance-associated module including six lncRNAs, three RBPs, and 58 mRNAs. Then, we applied WGCNA of the co-expression/regulatory networks to identify the potential molecular mechanisms of insulin resistance in patients with PCOS.

Using the IRLMN, we identified six hub lncRNAs (**Figure 3B**) that were highly related to insulin resistance in patients with PCOS. Then, we identified six other hub lncRNAs (**Table 2**) that were significantly related to insulin resistance in patients with PCOS *via* WGCNA. lncRNA RP11-151A6.4 was found to be the hub lncRNA in both IRLMN and WGCNA, indicating a greater role in patients with PCOS and insulin resistance. RP11-151A6.4 was found to be significantly higher in both subcutaneous and omental adipose tissues of patients with insulin resistance compared with healthy patients (control). The expression level of RP11-151A6.4 in ovarian granulosa cells was increased in patients with PCOS compared with control women and was also higher in obese women with PCOS and PCOS patients with hyperinsulinemia and higher HOMA-IR values. RP11-151A6.4 is an intronic lncRNA containing four exons. It is highly expressed in human testicular and adrenal tissues, which are responsible for androgen production (Roy et al., 2008; The GTEx Consortium, 2013; Chaoyong et al., 2014) (http://www.noncode.org//and http://genome.ucsc.edu/). PCOS is mainly a hyperandrogenic disorder. Androgen excess is not only a central mechanism in oligo-ovulation and cutaneous manifestations (hirsutism, acne, and alopecia) but also a major contributor to insulin resistance, metabolic dysfunction, and adipose tissue dysfunction in patients with PCOS (Escobar-Morreale and San Millan, 2007; Montes-Nieto et al., 2013; Dumesic et al., 2016). Thus, PCOS might be caused by a vicious circle of androgen contributing abdominal and visceral adipose tissue deposition, by inducing insulin resistance and compensatory hyperinsulinism, which further facilitates androgen excess by the ovaries and adrenal glands in patients with PCOS (Escobar-Morreale and San Millan, 2007; Escobar-Morreale, 2018). Consequently, RP11-151A6.4 might be a vital mediator for insulin resistance, androgen excess, and adipose tissue dysfunction in patients with PCOS.

Insulin has multiple functions beyond the regulation of glucose uptake. It affects metabolic processes and ovarian steroidogenesis, as well as ovulation and body weight in patients with PCOS (Diamanti-Kandarakis and Dunaif, 2012). Through GO enrichment analysis and pathway analysis for genes, we found that “regulation of metabolic process,” “insulin secretion,” “lipid metabolic process,” and “very long-chain fatty acid-CoA ligase activity” were significant and highly related to insulin resistance. Nevertheless, “cAMP signaling pathway” and “PI3K-Akt signaling pathway” were also significantly enriched in the modules.

There have been several studies about insulin action on lipid metabolism in patients with PCOS. Insulin resistance resulting from obesity, physical inactivity, or other clinical conditions might contribute to dyslipidemia, hyperglycemia, and hypertension (Lim et al., 2019). Changes in catecholamine regulation of lipolysis have also been reported in patients with PCOS (Ek et al., 1997; Ek et al., 2002). Sensitivity of catecholamine-stimulated lipolysis decreased in adipocytes isolated from the subcutaneous fat depot (Ek et al., 1997) and increased in adipocytes isolated from the visceral fat depot (Ek et al., 2002) in lean patients with PCOS. The alteration in catecholamine-stimulated lipolysis may contribute to hepatic insulin resistance by increasing portal delivery of free fatty acids to the liver (Cao et al., 2018). Some suggest that circulating insulin and androgens may have opposing effects on lipid profiles in patients with PCOS, particularly on the bioactive lipid metabolites derived from polyunsaturated fatty acids (Li et al., 2017). However, a study reported that there was no significant difference in lipid uptake between patients with PCOS and control myotubes (Cruz-Color et al., 2019).

Studies have shown that follicle-stimulating hormone triggers the cAMP/PKA pathway and pathways mediated by different kinases, such as PI3K and Akt/PKB. Thus, follicle-stimulating hormone may play a significant role in follicle maturation *via* the cAMP pathway (Zeleznik et al., 2003; Hunzicker-Dunn et al., 2012). The relationship between the cAMP pathway and follicle-stimulating hormone has been shown to induce insulin receptor substrate-2 expression in human and rat granulosa cells, thereby activating PI3K, Akt, and glucose uptake (Anjali et al., 2015).

Moreover, it has been reported that various serine/threonine kinases in the insulin signaling pathway, such as PI3K and Akt/PKB, phosphorylate the insulin receptor substrate to attenuate signaling, providing a feedback mechanism to terminate insulin action (Saltiel and Kahn, 2001; Draznin, 2006). Studies have suggested that a defect downstream of insulin receptor substrate-1 phosphorylation or PI3K activation is responsible for insulin resistance in some patients with PCOS (Cheatham and Kahn, 1995; Saltiel and Kahn, 2001; Diamanti-Kandarakis and Dunaif, 2012). However, some studies reported no differences in insulin-stimulated insulin receptor substrate-1-associated PI3K activity in the skeletal muscle in patients with PCOS, compared with the control (Dunaif et al., 2001; Hojlund et al., 2008).

The GSE8157 data from GEO contained 23 samples (10 cases and 13 controls), and we calculated the DEGs by the means of SAM. For WGCNA, some samples could not be well classified through cluster analysis. Thus, the outlier samples were deleted, and the remaining 13 samples (seven cases and six controls) were clustered again. Because of the deficiency and selection of samples, the results might elicit a false positive. More lncRNAs might have been annotated, if probe re-annotation was used in an exon microarray. In contrast, our microarray focused on testing mRNA expression; thus, fewer lncRNAs might have been found through probe re-annotation. Lastly, some genes were lost in the process of converting gene IDs from different databases, because of database discrepancies.

Nonetheless, our results showed for the first time the differences between lncRNA and mRNA profiles from healthy women and patients with PCOS and insulin resistance. We built a molecular interaction network linked to PCOS with insulin resistance and discovered 12 novel and hub lncRNAs correlated to insulin resistance in PCOS. Moreover, lncRNA RP11-151A6.4 might play a significant role in these processes. These potential molecular candidates might be important for future investigations and could elucidate the underlying mechanisms of insulin resistance.

## Data Availability

Data of RBP–lncRNA and RBP–mRNA interactions are publicly available at starBase V3.0 database (http://starbase.sysu.edu.cn/). Human long non-coding transcript sequences and protein-coding transcript sequences can be downloaded from the GENCODE database (https://www.gencodegenes.org). The two public gene expression datasets GSE8157 and GSE20950 can be downloaded from GEO database (https://www.ncbi.nlm.nih.gov/geo/). 

## Ethics Statement

The study was carried out in accordance with the recommendations of the ART Ethics Committee of Ren Ji Hospital, School of Medicine, Shanghai Jiao Tong University (number 2017041411) and was approved by said committee.

## Author Contributions

JZ and JH contributed equally to this work and authored this manuscript. Z-JC and YD contributed to the conception of the study. JZ and JH designed the study. JZ organized the database and performed the statistical analysis. JZ and JH wrote the first draft of the manuscript.. XG, WC, and SL revised the manuscript. All authors contributed to manuscript revision and read and approved the submitted version.

## Funding

This work was partly supported by grants from the National Key Research and Development Program of China (Nos. 2018YFC1003202 and 2017YFC1001002), National Natural Science Foundation (No. 81671414 , 81490743 and 81671413), and Shanghai Commission of Science and Technology (No. 17DZ2271100).

## Conflict of Interest Statement

The authors declare that the research was conducted in the absence of any commercial or financial relationships that could be construed as a potential conflict of interest.

## Supplementary Material

Supplementary Figure 1 Principal Components Plot analysis on GSE8157. The principal components plot analysis (PCA) result is consistent with the clustering analysis; The samples of PCOS and Control were not clustering well. 0 represented Control (red dots); 1 represented PCOS (blue rectangles).

## References

[B1] Adashi E. Y. Hsueh A. J. Yen S. S. (1981). Insulin enhancement of luteinizing hormone and follicle-stimulating hormone release by cultured pituitary cells. Endocrinology 108, 1441–1449. 10.1210/endo-108-4-1441 6781875

[B2] Anithasri A. Ananthanarayanan P. H. Veena P. (2019). A study on omentin-1 and prostate specific antigen in women on treatment for polycystic ovary syndrome. Indian. J. Clin. Biochem. 34, 108–114. 10.1007/s12291-017-0723-9 30728681 PMC6346616

[B3] Anjali G. Kaur S. Lakra R. Taneja J. Kalsey G. S. Nagendra A. (2015). FSH stimulates IRS-2 expression in human granulosa cells through cAMP/SP1, an inoperative FSH action in PCOS patients. Cell. Signal. 27, 2452–2466. 10.1016/j.cellsig.2015.09.011 26388164

[B4] Barbieri R. L. Makris A. Randall R. W. Daniels G. Kistner R. W. Ryan K. J. (1986). Insulin stimulates androgen accumulation in incubations of ovarian stroma obtained from women with hyperandrogenism. J. Clin. Endocrinol. Metab. 62, 904–910. 10.1210/jcem-62-5-904 3514651

[B5] Cao L. Wang Z. Wan W. (2018). Suppressor of cytokine signaling 3: emerging role linking central insulin resistance and Alzheimer’s disease. Front. Neurosci. 12, 417. 10.3389/fnins.2018.00417 29973864 PMC6020761

[B6] Chaoyong X. Jiao Y. Hui L. Ming L. Guoguang Z. Dechao B. (2014). NONCODEv4: exploring the world of long non-coding RNA genes. Nucleic Acids Res. 42, 98–103. 10.1093/nar/gkt1222 PMC3965073 24285305

[B7] Cheatham B. Kahn C. R. (1995). Insulin action and the insulin signaling network. Endocr. Rev. 16, 117–142. 10.1210/edrv-16-2-117 7781591

[B8] Corkey B.E. (2011). Banting Lecture 2011: hyperinsulinemia: cause or consequence? Diabetes 61, 4–13. 10.2337/db11-1483 PMC3237642 22187369

[B9] Cruz-Color L. Hernandez-Nazara Z. H. Maldonado-Gonzalez M. Navarro-Muniz E. Dominguez-Rosales J. A. Torres-Baranda J. R. (2019). Association of the PNPLA2, SCD1 and leptin expression with fat distribution in liver and adipose tissue from obese subjects. Exp. Clin. Endocrinol. Diabetes 5, e14469. 10.1055/a-0829-6324 30754064

[B10] Diamanti-Kandarakis E. Dunaif A. (2012). Insulin resistance and the polycystic ovary syndrome revisited: an update on mechanisms and implications. Endocr. Rev. 33, 981–1030. 10.1210/er.2011-1034 23065822 PMC5393155

[B11] Draznin B. (2006). Molecular mechanisms of insulin resistance: serine phosphorylation of insulin receptor substrate-1 and increased expression of p85alpha: the two sides of a coin. Diabetes 55, 2392–2397. 10.2337/db06-0391 16873706

[B12] Dumesic D. A. Akopians A. L. Madrigal V. K. Ramirez E. Margolis D. J. Sarma M. K. (2016). Hyperandrogenism accompanies increased intra-abdominal fat storage in normal weight polycystic ovary syndrome women. J. Clin. Endocrinol. Metab. 101, 4178–4188. 10.1210/jc.2016-2586 27571186 PMC5095243

[B13] Dumesic D. A. Abbott D. H. (2008). Implications of polycystic ovary syndrome on oocyte development. Semin. Reprod. Med. 26, 53–61. 10.1055/s-2007-992925 18181083 PMC2655636

[B14] Dunaif A. Wu X. Lee A. Diamanti-Kandarakis E. (2001). Defects in insulin receptor signaling *in vivo* in the polycystic ovary syndrome (PCOS). Am. J. Physiol. Endocrinol. Metab. 281, E392–E399. 10.1152/ajpendo.2001.281.2.E392 11440917

[B15] Ek I. Arner P. Bergqvist A. Carlstrom K. Wahrenberg H. (1997). Impaired adipocyte lipolysis in nonobese women with the polycystic ovary syndrome: a possible link to insulin resistance? J. Clin. Endocrinol. Metab. 82, 1147–1153. 10.1210/jcem.82.4.3899 9100587

[B16] Ek I. Arner P. Ryden M. Holm C. Thorne A. Hoffstedt J. (2002). A unique defect in the regulation of visceral fat cell lipolysis in the polycystic ovary syndrome as an early link to insulin resistance. Diabetes 51, 484–492. 10.2337/diabetes.51.2.484 11812759

[B17] Escobar-Morreale H. F. (2018). Polycystic ovary syndrome: definition, aetiology, diagnosis and treatment. Nat. Rev. Endocrinol. 14, 270–284. 10.1038/nrendo.2018.24 29569621

[B18] Escobar-Morreale H. F. San Millan J. L. (2007). Abdominal adiposity and the polycystic ovary syndrome. Trends Endocrin. Met. 18, 266–272. 10.1016/j.tem.2007.07.003 17693095

[B19] Goyal N. Kesharwani D. Datta M. (2018). Lnc-ing non-coding RNAs with metabolism and diabetes: roles of lncRNAs. Cell. Mol. Life Sci. 75, 1827–1837. 10.1007/s00018-018-2760-9 29387902 PMC11105777

[B20] Hardy O. T. Perugini R. A. Nicoloro S. M. Gallagher-Dorval K. Puri V. Straubhaar J. (2011). Body mass index-independent inflammation in omental adipose tissue associated with insulin resistance in morbid obesity. Surg. Obes. Relat. Dis. 7, 60–67. 10.1016/j.soard.2010.05.013 20678967 PMC2980798

[B21] Hojlund K. Glintborg D. Andersen N. R. Birk J. B. Treebak J. T. Frosig C. (2008). Impaired insulin-stimulated phosphorylation of Akt and AS160 in skeletal muscle of women with polycystic ovary syndrome is reversed by pioglitazone treatment. Diabetes 57, 357–366. 10.2337/db07-0706 17977950

[B22] Hunzicker-Dunn M. E. Lopez-Biladeau B. Law N. C. Fiedler S. E. Carr D. W. Maizels E. T. (2012). PKA and GAB2 play central roles in the FSH signaling pathway to PI3K and AKT in ovarian granulosa cells. Proc. Natl. Acad. Sci. U S A 109, E2979–E2988. 10.1073/pnas.1205661109 23045700 PMC3497822

[B23] Jiao J. Shi B. Wang T. Fang Y. Cao T. Zhou Y. (2018). Characterization of long non-coding RNA and messenger RNA profiles in follicular fluid from mature and immature ovarian follicles of healthy women and women with polycystic ovary syndrome. Hum. Reprod. 33, 1735–1748. 10.1093/humrep/dey255 30052945

[B24] Jun Z. Jieying X. Wangshen W. Han Z. Hongbin L. Xiaojing L. (2018). Long non-coding RNA LINC-01572:28 inhibits granulosa cell growth *via a* decrease in p27 (Kip1) degradation in patients with polycystic ovary syndrome. EBioMedicine 36, 536–538. 10.1016/j.ebiom.2018.09.043 PMC6197751 30293818

[B25] Langfelder P. Horvath S. (2008). WGCNA: an R package for weighted correlation network analysis. BMC Bioinform. 9, 559. 10.1186/1471-2105-9-559 PMC2631488 19114008

[B26] Li C. Li X. Miao Y. Wang Q. Jiang W. Xu C. (2009). SubpathwayMiner: a software package for flexible identification of pathways. Nucleic Acids Res. 37, e131. 10.1093/nar/gkp667 19706733 PMC2770656

[B27] Li S. Chu Q. Ma J. Sun Y. Tao T. Huang R. (2017). Discovery of novel lipid profiles in PCOS: do insulin and androgen oppositely regulate bioactive lipid production? J. Clin. Endocrinol. Metab. 102, 810–821. 10.1210/jc.2016-2692 27886515 PMC5477809

[B28] Lim S. S. Kakoly N. S. Tan J. Fitzgerald G. Bahri K. M. Joham A. E. (2019). Metabolic syndrome in polycystic ovary syndrome: a systematic review, meta-analysis and meta-regression. Obes. Rev. 20, 339–352. 10.1111/obr.12762 30339316

[B29] Matthews D. R. Hosker J. P. Rudenski A. S. Naylor B. A. Treacher D. F. Turner R. C. (1985). Homeostasis model assessment: insulin resistance and beta-cell function from fasting plasma glucose and insulin concentrations in man. Diabetologia 28, 412–419 10.1007/BF00280883 3899825

[B30] Miller W. P. Ravi S. Martin T. D. Kimball S. R. Dennis M. D. (2017). Activation of the stress response kinase JNK (c-Jun N-terminal kinase) attenuates insulin action in retina through a p70S6K1-dependent mechanism. J. Biol. Chem. 292, 1591–1602. 10.1074/jbc.M116.760868 27965359 PMC5290937

[B31] Montes-Nieto R. Insenser M. Martinez-Garcia M. A. Escobar-Morreale H. F. (2013). A nontargeted proteomic study of the influence of androgen excess on human visceral and subcutaneous adipose tissue proteomes. J. Clin. Endocrinol. Metab. 98, E576–E585. 10.1210/jc.2012-3438 23348399

[B32] Morley L. C. Tang T. Yasmin E. Norman R. J. Balen A. H. (2017). Insulin-sensitising drugs (metformin, rosiglitazone, pioglitazone, D-chiro-inositol) for women with polycystic ovary syndrome, oligo amenorrhoea and subfertility. Cochrane Database Syst. Rev. 11, CD003053. 10.1002/14651858.CD003053.pub6 29183107 PMC6486196

[B33] Nestler J. E. Jakubowicz D. J. de Vargas A. F. Brik C. Quintero N. Medina F. (1998). Insulin stimulates testosterone biosynthesis by human thecal cells from women with polycystic ovary syndrome by activating its own receptor and using inositolglycan mediators as the signal transduction system. J. Clin. Endocrinol. Metab. 83, 2001–2005. 10.1210/jcem.83.6.4886 9626131

[B34] Nestler J. E. Powers L. P. Matt D. W. Steingold K. A. Plymate S. R. Rittmaster R. S. (1991). A direct effect of hyperinsulinemia on serum sex hormone-binding globulin levels in obese women with the polycystic ovary syndrome. J. Clin. Endocrinol. Metab. 72, 83–89. 10.1210/jcem-72-1-83 1898744

[B35] Quinn J. J. Chang H. Y. (2016). Unique features of long non-coding RNA biogenesis and function. Nat. Rev. Genet. 17, 47–62. 10.1038/nrg.2015.10 26666209

[B36] Ricardo Azziz E. C. Z. C. Richard S. Legro D. L. B. N. Yildiz A. B. O. (2016). Polycystic ovary syndrome. Nat. Rev. Dis. Primers 2, 16057 10.1038/nrdp.2016.58 27510637

[B37] Rotterdam E. A. P. (2004). Revised 2003 consensus on diagnostic criteria and long-term health risks related to polycystic ovary syndrome. Fertil. Steril. 81, 19–25. 10.1016/j.fertnstert.2003.10.004 14711538

[B38] Roy P. Alevizaki M. Huhtaniemi I. (2008). *In vitro* bioassays for androgens and their diagnostic applications. Hum. Reprod. Update 14, 73–82. 10.1093/humupd/dmm038 18056750

[B39] Saltiel A. R. Kahn C. R. (2001). Insulin signalling and the regulation of glucose and lipid metabolism. Nature 414, 799–806. 10.1038/414799a 11742412

[B40] Skov V. Glintborg D. Knudsen S. Tan Q. Jensen T. Kruse T. A. (2008). Pioglitazone enhances mitochondrial biogenesis and ribosomal protein biosynthesis in skeletal muscle in polycystic ovary syndrome. PLoS One 3, e2466. 10.1371/journal.pone.0002466 18560589 PMC2413008

[B41] The GTEx Consortium, and 2013 The Genotype-Tissue Expression (GTEx) project. Nat. Genet. 45, 580–585. 10.1038/ng.2653 23715323 PMC4010069

[B42] Tosi F. Negri C. Brun E. Castello R. Faccini G. Bonora E. (2011). Insulin enhances ACTH-stimulated androgen and glucocorticoid metabolism in hyperandrogenic women. Eur. J. Endocrinol. 164, 197–203. 10.1530/EJE-10-0782 21059865

[B43] Wang C. Shen F. Zhu Y. Fang Y. Lu S. (2017). Telomeric repeat-containing RNA (TERRA) related to polycystic ovary syndrome (PCOS). Clin. Endocrinol. 86, 552–559. 10.1111/cen.13283 27864985

[B44] WHO Expert Consultation, and 2004 Appropriate body-mass index for Asian populations and its implications for policy and intervention strategies. Lancet 363, 157–163. 10.1016/S0140-6736(03)15268-3 14726171

[B45] Zeleznik A. J. Saxena D. Little-Ihrig L. (2003). Protein kinase B is obligatory for follicle-stimulating hormone-induced granulosa cell differentiation. Endocrinology 144, 3985–3994. 10.1210/en.2003-0293 12933673

[B46] Zhang B. Horvath S. (2005). A general framework for weighted gene co-expression network analysis. Stat. Appl. Genet. Mol. Biol. 4, 1–43. 10.2202/1544-6115.1128 16646834

