# Supplementary figures and images for: Polycystic Ovary Syndrome: Novel and Hub lncRNAs in the Insulin Resistance-Associated lncRNA–mRNA Network
Source: Front Genet. 2019 Aug 22;10:772. doi: 10.3389/fgene.2019.00772 (PMC6715451; doi:10.3389/fgene.2019.00772)

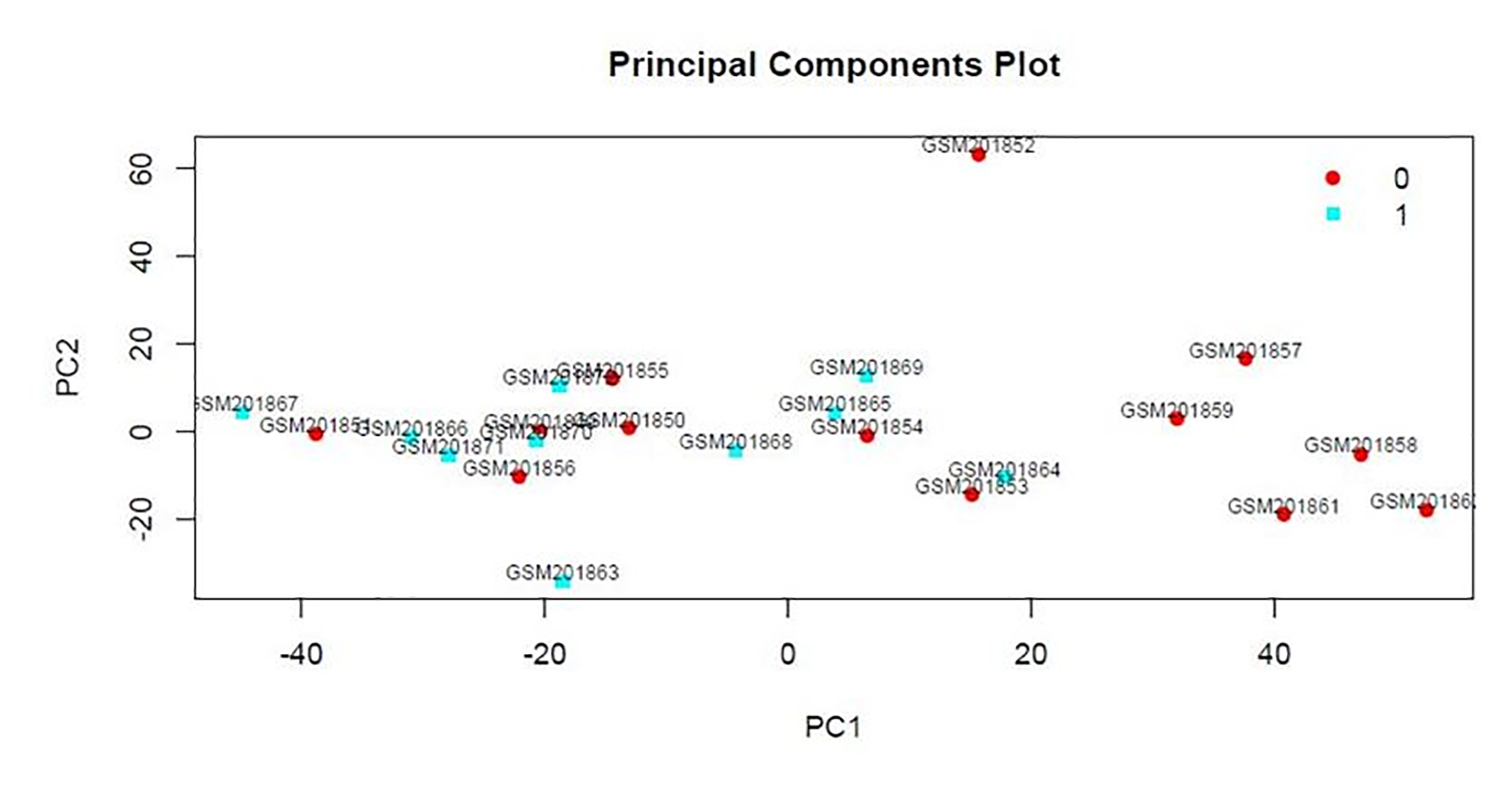

Supplement: Supplementary Figure 1 — Principal Components Plot analysis on GSE8157. The principal components plot analysis (PCA) result is consistent with the clustering analysis; The samples of PCOS and Control were not clustering well. 0 represented Control (red dots); 1 represented PCOS (blue rectangles). [file Image_1.jpg]
