# Supplementary material for: Polycystic Ovary Syndrome: Novel and Hub lncRNAs in the Insulin Resistance-Associated lncRNA–mRNA Network
Source: Front Genet. 2019 Aug 22;10:772. doi: 10.3389/fgene.2019.00772 (PMC6715451; doi:10.3389/fgene.2019.00772)
Supplement: Supplementary file 2 [file Table_1.docx]

| **Primer** | 5' to 3' |
| --- | --- |
| RP11-151A6.4-F | AATGACCATTGCGTGCTGAG |
| RP11-151A6.4-R | AGGCACTCTCCCTCAGAAAC |

Supplemental Table S1. The primer sequences of the tested genes (RP11-151A6.4)
