# Supplementary material for: Polycystic Ovary Syndrome: Novel and Hub lncRNAs in the Insulin Resistance-Associated lncRNA–mRNA Network
Source: Front Genet. 2019 Aug 22;10:772. doi: 10.3389/fgene.2019.00772 (PMC6715451; doi:10.3389/fgene.2019.00772)
Supplement: Supplementary file 3 [file Table_2.docx]

Supplemental Table S2: Range of HOMA-IR in patients with PCOS

| HOMA-IR | | |
| --- | --- | --- |
| Mean | | 4.92 |
| Standard deviation | | 0.70 |
| Standard error | | 4.27 |
| Minimum | | 0.95 |
| Maximum | | 20.73 |
| Tertile | 33.33 | 2.79 |
|  | 66.66 | 4.49 |
