# Supplementary material for: Polycystic Ovary Syndrome: Novel and Hub lncRNAs in the Insulin Resistance-Associated lncRNA–mRNA Network
Source: Front Genet. 2019 Aug 22;10:772. doi: 10.3389/fgene.2019.00772 (PMC6715451; doi:10.3389/fgene.2019.00772)
Supplement: Supplementary file 4 [file Table_3.docx]

Table 3: Background information of patients with polycystic ovary syndrome (PCOS) and controls

|  | **PCOS (n = 52)** | **Control (n = 42)** | **P value** |
| --- | --- | --- | --- |
| **Age (years)** | 28.23 ± 3.47 | 28.83 ± 2.86 | 0.345 |
| **BMI (kg/m^2^)^a^** | 25.92 ± 3.58 | 22.19 ± 2.92 | < 0.0001 |
| **Basal FSH(IU/L)^b^** | 5.59 ± 1.00 | 6.30 ± 1.37 | < 0.01 |
| **Basal LH(IU/L)^a^** | 9.64 ± 4.57 | 5.12 ± 1.92 | < 0.0001 |
| **Basal T(ng/dL)^a^** | 42.28 ± 21.50 | 20.06 ± 6.95 | < 0.0001 |
| **AMH (ng/ml)^a^** | 9.74 ± 4.15 | 5.09 ± 3.13 | < 0.0001 |
| **Fasting glucose (mmol/L)^c^** | 5.59 ± 0.52 | 5.35 ± 0.35 | 0.022 |
| **Fasting insulin (mIU/L)^b^** | 20.57 ± 16.71 | 9.31 ± 3.53 | < 0.01 |
| **HOMA-IR^a^** | 4.92 ± 4.27 | 1.70 ± 1.23 | < 0.0001 |

All data are mean ± SD value. a : p<0.001, b : p<0.01, c : p<0.05; Follicle-stimulating hormone (FSH), luteinizing hormone (LH), testosterone (T), anti-Müllerian hormone (AMH).
